# Case Report: When multiple system atrophy masquerades as CASPR2 autoimmune encephalopathy: a diagnostic pitfall

**DOI:** 10.3389/fnhum.2025.1658933

**Published:** 2025-09-15

**Authors:** Jun Zhang, Yueming Wang, Le Chen, Jun Li

**Affiliations:** ^1^Clinical Research Center, Xuanwu Hospital Capital Medical University, Beijing, China; ^2^Department of Neurology, Beijing Tsinghua Changgung Hospital, School of Clinical Medicine, Tsinghua Medicine, Tsinghua University, Beijing, China

**Keywords:** multiple system atrophy, CASPR2 autoimmune encephalopathy, cerebellum atrophy, ataxia, EAS-EMG

## Abstract

**Introduction:**

Multiple system atrophy (MSA) is a sporadic, adult-onset neurodegenerative disorder characterized by rapid progression. Early diagnosis remains particularly challenging, especially when CASPR2 antibodies are detected during the early stages of disease progression.

**Case presentation:**

A 56-year-old female presented with chronic progressive cerebellar ataxia. Serum analysis revealed the presence of CASPR2 antibodies. Brain MRI demonstrated atrophy of the brainstem and cerebellum. The external anal-sphincter electromyography (EAS-EMG) indicated autonomic nerve dysfunction in the absence of overt clinical symptoms. Despite treatment with high-dose corticosteroids and immunosuppressants, no clinical improvement was observed. During follow-up, symptoms of autonomic nerve dysfunction emerged, leading to a clinical established diagnosis of cerebellar subtype of multiple system atrophy (MSA-C).

**Conclusion:**

In the early stages of MSA, autonomic nerve dysfunction may not manifest clinically. Therefore, close follow-up is essential for accurate diagnosis. EAS-EMG exhibits high sensitivity in detecting subclinical autonomic dysfunction. Although the co-occurrence of MSA-C and high-titer (1:100) CASPR2-IgG is uncommon, this case highlights the critical importance of relying on the overall clinical presentation, rather than on isolated laboratory findings, to establish the primary diagnosis.

## Introduction

Multiple system atrophy (MSA) is a sporadic, rapidly progressive neurodegenerative disorder with adult-onset, primarily affecting individuals in their 50s and 60s (Miki et al., 2019). The pathogenic hallmark of MSA is the aggregation of insoluble α-synuclein, predominantly in oligodendrocytes (Enomoto et al., 2025). Clinically, MSA is characterized by autonomic dysfunction, poorly levodopa-responsive parkinsonism, and cerebellar ataxia. Based on clinical features, MSA is categorized into two subtypes: the parkinsonian form (MSA-P), associated with striatonigral degeneration, and the cerebellar form (MSA-C), linked to olivopontocerebellar atrophy (Fanciulli and Wenning, 2015). Patients with MSA-C typically present with symptoms such as dysarthria, movement and coordination abnormalities, and visual or swallowing difficulties (Lim et al., 2025). Prior studies have shown that 73% of individuals with MSA exhibit initial autonomic dysfunction symptoms (Yamamoto et al., 2014). Furthermore, approximately 60% of MSA patients experience urinary symptoms either before or concurrently with motor disturbances (Sakakibara et al., 2000). These characteristics contribute to a higher likelihood of misdiagnosis in the early stages, particularly in cases of isolated ataxia.

Contactin-associated protein-like 2 (CASPR2) is a transmembrane cell adhesion protein expressed in neurons of both the central and peripheral nervous systems. CASPR2 antibody-associated neurological diseases exhibit a broad clinical spectrum, which can be categorized into three overlapping autoimmune syndromes: CASPR2 limbic encephalitis, Morvan syndrome, and Isaacs syndrome. These diseases typically manifest in individuals aged ≥50 years and are more prevalent in males (Joubert, 2024). Notably, the association between nonparaneoplastic cerebellar ataxia and CASPR2 antibodies was first documented in 2012 (Becker et al., 2012), with cerebellar symptoms estimated to occur in approximately 14% of patients with anti-CASPR2 antibodies (Boyko et al., 2020). In the case series reported by Becker et al. (2012), four out of six patients (66.7%) presented with isolated cerebellar ataxia, while two patients (33.3%) exhibited an insidious onset with progressive deterioration. Consequently, these cases may initially be misdiagnosed as MSA-C. Herein, this report describes a rare and clinically challenging case of a patient with MSA-C and an incidental high-titer (1:100) CASPR2-IgG seropositivity. It underscores the necessity of integrating the overall clinical presentation, rather than relying solely on isolated laboratory results in determining the primary diagnosis.

## Case report

A 56-year-old female was admitted to our hospital for evaluation of an uncoordinated gait and slurred speech. Her symptoms began insidiously over a 12-month period and progressed chronically. At the 9 months after disease onset, her symptoms worsened to the extent that her family could no longer understand her speech, and she required assistance from family members to walk. She reported no issues with defecation or urination, and her weight remained stable. However, she had experienced rapid eye movement sleep behavior disorder (RBD) for 6 years which was diagnosed in another hospital. She denied any family history of ataxia, particularly genetic diseases or progressive neurological disorders, and had no history of tobacco or alcohol use or long-term medication. Then, she was hospitalized in the Neurological Department of Beijing Tsinghua Changgung Hospital for further evaluation.

On admission, the patient exhibited several notable clinical findings, including dysarthria, exaggerated bilateral tendon reflexes, positive bilateral Babinski signs and Chaddock signs, dysmetria during finger-to-nose and heel–knee-shin tests, a wide-based gait, and impaired tandem gait. The Romberg test revealed instability both with eyes open and closed. Notably, there were no significant changes in blood pressure while the patient was in standing, seated, or supine positions. The muscle strength of the limbs was normal.

Laboratory tests revealed positive anti-nuclear antibodies (titer 1:320) and positive anti-SSA antibodies, although the patient reported no related symptoms. Serum and cerebrospinal fluid (CSF) autoimmune encephalitis-associated antibody testing (NMDA-R-Ab, CASPR2-Ab, AMPA1-Ab, AMPA2-Ab, LGI1-Ab, GABAB-R-Ab, GAD65-Ab) and paraneoplastic syndromes (CV2/CRMP5, Ma2/Ta, Ri, Yo, Hu, Amphiphysin) were sent to the Peking Union Medical College Hospital. The anti-CASPR2 antibodies was positive (IgG titer 1:100, fixed CBA) in serum, while evaluations for other antibodies were negative in both serum and CSF. Routine serological tests, including liver and renal function, thyroid function, vitamin B12 and folic acid levels, anti-thyroid antibodies, anti-neutrophil cytoplasmic antibodies, and rheumatoid factor, were all within normal limits. Tests for hepatitis, syphilis, and human immunodeficiency virus (HIV) were negative. Cerebrospinal fluid (CSF) analysis demonstrated normal white blood cell count, protein levels, glucose, immunoglobulins, and intrathecal immunoglobulin synthesis rates. The oligoclonal bands were absent. CSF screening for bacterial and viral pathogens was negative. Tumor markers in the serum were negative, and genetic testing for spinocerebellar ataxia revealed no abnormalities.

**Figure 1:**
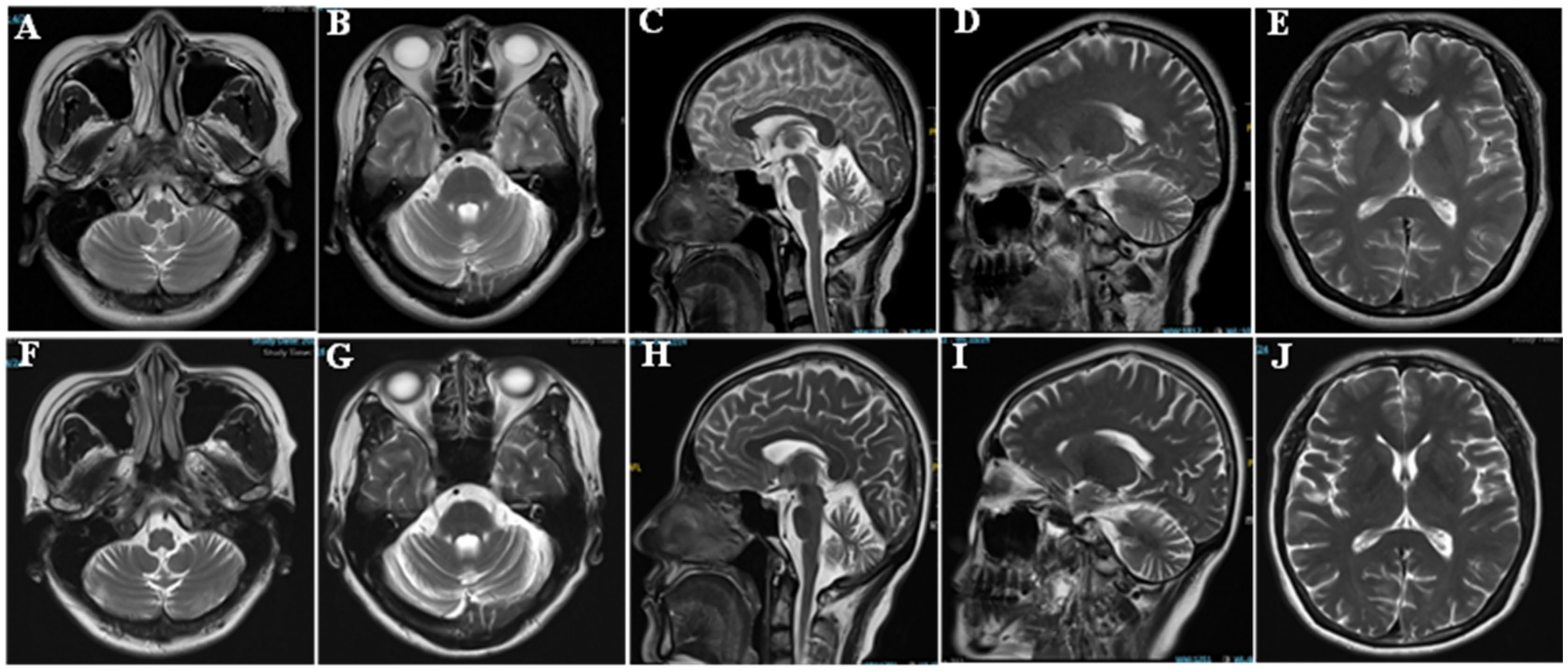
Brain imaging of the patient. **(A,B,E,F,G,J)** Axial T2 weighted image. **(C,D,H,I)** Sagittal T2 weighted images.

**Figure 2:**
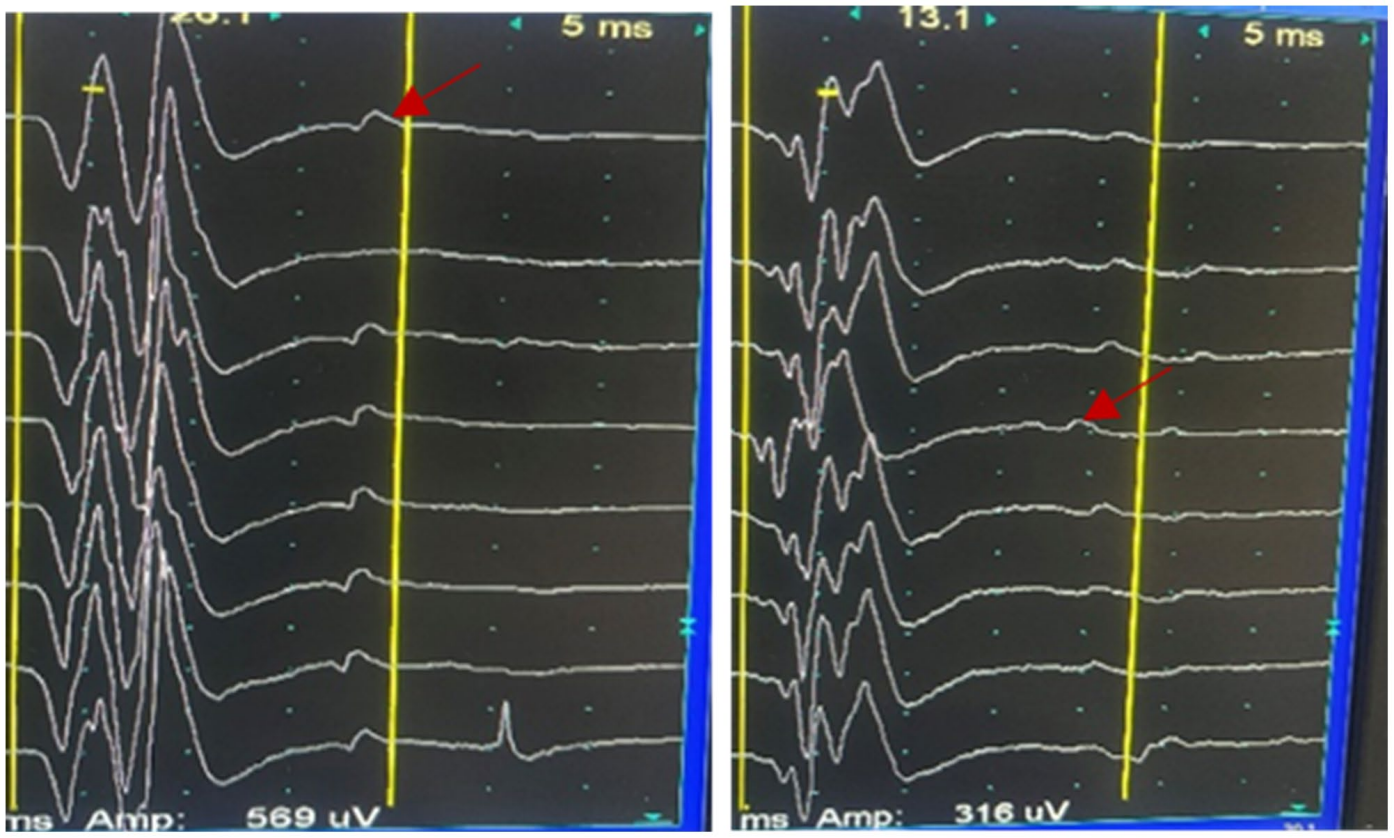
Satellite potential was observed (the red arrow) in the EAS-EMG.

Her brain Magnetic Resonance Imaging (MRI) demonstrated atrophy in the brainstem and cerebellum and the putamen was normal (Figures 1A–E). The external anal-sphincter electromyography (EAS-EMG) exhibited increased duration of motor unit action potential (MUAP) and satellite potential was observed (Figure 2; Table 1). The bladder ultrasonography for residual urine were both normal. To rule out an underlying neoplasm, computed tomography (CT) scan of the chest, abdomen, and pelvis were performed, with no significant findings.

**Table 1:** The EAS-EMG parameters of this patient.

Muscle	Satellite potential	Spontaneous activity			Volitional MUAPs			Max volitional activity
		Fibs	+Wave	Fasc	Dur (ms)	Amp (uv)	Ploy (%)	
Sphincter anal L	15/30	2+	2+	–	14.3	336	45.2	Contraction weakness
Sphincter anal R	14/29	1+	3+	–	14.4	177	26.7	Contraction weakness

Then, CASPR2 Autoimmune Encephalopathy was the initially suspected diagnosis and she received treatment consisting of intravenous methylprednisolone (1 g daily for 3 days, followed by 500 mg daily for 3 days), oral prednisolone (1 mg/kg), and Mycophenolate Mofetil capsules (750 mg twice daily). Despite this regimen, her symptoms remained stable initially but subsequently worsened progressively. Approximately 3 months after discharge, she began to have symptoms of autonomic nervous system dysfunction such as frequent urination and constipation. Additionally, there was a 30 mmHg difference in systolic blood pressure between the supine and standing positions, indicative of orthostatic hypotension. Six months after discharge, we retested the serum anti-CASPR2 antibody level (Peking Union Medical College Hospital), which had decreased to 1:10 (fixed CBA).

However, her gait disorder continued to deteriorate, resulting in frequent falls and ultimately leading to a hip fracture 1 year after discharge. At 1 year follow-up visit, the patient was in a wheelchair and unable to walk because of the fracture. The patient’s speech articulation became more unclear, and the finger-to-nose movement of both upper limbs was even more unstable. The difference in blood pressure between the supine and standing positions was >30 mmHg. Repeat head MRI demonstrated progressive cerebellar and brainstem atrophy (Figures 1F–J). Based on these findings and persistent clinical features, the final diagnosis was revised to MSA-C (Wenning et al., 2022).

## Discussion

To our knowledge, reports of incidental high-titer CASPR2 positivity in patients with otherwise typical MSA-C are exceedingly rare. This case serves as a critical educational example of a potential diagnostic pitfall. In the early stage of the patient’s illness, due to the absence of definitive diagnostic evidence for MSA, and given a previous case report describing isolated cerebellar ataxia with a CASPR2 antibody titer of 1:100 (Chen et al., 2024), clinicians initially leaned toward considering CASPR2-related diseases as a possible diagnosis based on clinical manifestations and epidemiological characteristics. But the unexplained bilateral Babinski sign is still the argument. It is important to note that CASPR2-related diseases are treatable conditions with higher intervention value compared to MSA. However, following immunotherapy, neither the clinical symptoms nor cranial MRI imaging showed significant improvement. As the disease progressed, symptoms of autonomic dysfunction gradually manifested. Despite a high-titer (1:100) CASPR2-IgG in serum, the final diagnosis is MSA-C, based on established diagnostic criteria and the overall clinical picture. The patient meets the criteria for “clinically established MSA” as outlined (Wenning et al., 2022). First, the disease followed a progressive neurodegenerative course with continuous decline over 2 years, unlike the fluctuating course typical of autoimmune encephalopathy. Second, the patient showed severe autonomic failure marked by early urinary incontinence and orthostatic hypotension which are core signs of MSA. Third, the main motor symptoms were those of a sporadic adult-onset cerebellar syndrome, including gait and limb ataxia, and cerebellar dysarthria. These three features form the core diagnostic criteria. Additional signs included an unexplained Babinski sign and rapid motor and speech decline. Brain MRI showed significant cerebellar atrophy, a hallmark of MSA-C. Importantly, there was no response to immunosuppressive therapy, and no signs of CASPR2-related features such as neuromyotonia, neuropathic pain, or limbic encephalitis. Thus, despite CASPR2 antibody positivity, the full clinical evidence supports MSA-C. This highlights the importance of interpreting lab results within the full clinical context.

In this case, symptoms such as frequent urination, constipation, and neurogenic orthostatic hypotension appeared about 1 year after motor symptoms began. This highlights the need for clinicians to remain vigilant, as autonomic involvement in MSA may initially be asymptomatic. Autonomic dysfunction is a common feature of MSA and CASPR2-associated disorders like Morvan syndrome (Joubert, 2024). In our patient, the autonomic dysfunction was consistent with the classic MSA phenotype. The absence of neuromyotonia, neuropathic pain, severe insomnia, or cognitive and psychiatric symptoms argued against Morvan’s syndrome or a primary CASPR2-associated autonomic neuropathy (Masrori et al., 2021). Nonetheless, this case highlights the importance of considering autoimmune mimics, including CASPR2 autoimmunity, in patients presenting with cerebellar ataxia and autonomic failure, and emphasizes the need for comprehensive antibody testing in atypical presentations. The differences between MSA and CASPR2-antibody associated disease were detailed in Table 2 (Wenning et al., 2022; Rubio-Agusti et al., 2011; Boyko et al., 2020; Joubert, 2024; Qin et al., 2021).

**Table 2:** The difference between MSA and CSPR2-Ab associated disease.

	MSA	CASPR2-Ab associated disease
Epidemiologic features	Sporadic, progressive adult (>30 years) onset, neurodegenerative disease	Sub-acute or insidious onset, predominance in older male patients (median age at onset of 65 years), autoimmune disorder
Core clinical features	1. Poorly L-dopa-responsive parkinsonism 2. Cerebellar syndrome 3. Autonomic dysfunction a. Unexplained voiding difficulties b. Unexplained urinary urge incontinence c. Neurogenic OH within 3 min of standing or head-up tilt test	1. Limbic system symptoms (cognitive impairment, seizures, etc.) 2. Cerebellar ataxia 3. Peripheral nerve hyperexcitability 4. Autonomic dysfunction (arrhythmia, urinary and fecal dysfunction, edema, itching, excessive sweating, excessive salivation and tearing) 5. Neuropathic pain 6. Weight loss 7. Insomia
Supportive clinical features	*Motor features* 1. Rapid progression within 3 years of motor onset 2. Moderate to severe postural instability within 3 years of motor onset 3. Craniocervical dystonia induced or exacerbated by L-dopa in the absence of limb dyskinesia 4. Severe speech impairment within 3 years of motor onset 5. Severe dysphagia within 3 years of motor onset 6. Unexplained Babinski sign 7. Jerky myoclonic postural or kinetic tremor 8. Postural deformities *Non-motor features* Stridor, Inspiratory sighs, Cold discolored hands and feet, Erectile dysfunction, Pathologic laughter or crying	1. Autoimmune limbic encephalitis 2. Isaacs syndrome (peripheral nerve hyperexcitability with cramps and fasciculations, neurogenic pain, dysautonomia) 3. Morvan syndrome (peripheral nerve hyperexcitability, severe insomnia, dysautonomia, dream-like enactment behavior, and visual hallucinations)
Key examination	Cranial MRI: Atrophy of Putamen (and signal decrease on iron-sensitive sequences), Middle cerebellar peduncle, Pons, Cerebellum 2. “Hot cross bun” sign 3. Increased diffusivity of Putamen or Middle cerebellar peduncle	1. Serum and/ or CSF CASPR2-Ab: positive (CBA), high titer (≥1:100) is highly specific 2. EMG: spontaneous neuromyotonia discharges, myoclonic potentials, spastic discharges, fasciculation potentials 3. Cranial MRI:normal, or high signal in the medial temporal lobe on T2-weighted imaging, cortical atrophy, meningeal enhancement changes 4. EEG: not specific, epileptic waves can be observed sometimes.
Exclusion criteria	1. Dopamine drugs are significantly and consistently effective 2. Unexplained olfactory decline during test 3. Cognitive fluctuations with significant changes in attention and alertness, and early decline in visual perception ability 4. Recurrent visual hallucinations induced by non-pharmacological factors within 3 years of disease onset 5. Dementia meets the DSM-V within 3 years of disease onset 6. Downgaze supranuclear palsy or slowing of vertical saccade movement 7. Brain MRI findings suggestive of an alternative diagnosis 8. Documentation of other causes that lead to autonomic failure, ataxia, or parkinsonism and resemble the patient’s symptoms	1. The brain MRI shows characteristic changes of MSA. 2. A typical progressive neurodegenerative disease course, without fluctuations or remissions. 3. Has no response to immunotherapy (requiring thorough trial before proceeding).
Diagnosis	Clinical features + supportive features +MRI marker + excluding others	Clinical features + excluding others + specific antibody testing
Treatment and outcome	*Treatment:* No disease-modifying treatment, only symptomatic supportive treatment. *Outcome:* Continues to progress rapidly, with a poor prognosis. The median survival period is approximately 6–10 years.	*Treatment:* No clinical guidelines or standard treatment protocols. First-line immunotherapy (glucocorticoids, IVIG, plasma exchange). Second-line immunosuppressants (cyclophosphamide, mycophenolate mofetil, etc.). *Outcome:* Most patients respond well to the treatment, some may experience recurrence.

MSA progression typically starts in the sacral spinal cord and spreads to other areas, causing movement disorders and cardiovascular autonomic dysfunction (Panicker et al., 2020). Damage to Onuf’s nucleus in the anterior sacral horn results in neurogenic changes on EAS-EMG, including prolonged motor unit action potential duration, increased polyphasic waveforms, and spontaneous or satellite potentials (Miao et al., 2020). Electrophysiological abnormalities appear early in the disease course, showing that EAS-EMG is highly sensitive for diagnosing MSA. When neurons in Onuf’s nucleus degenerate, the anal sphincter muscle fibers they innervate undergo denervation. Neuroreinnervation occurs gradually, but regenerated nerves are functionally unstable, resulting in satellite potentials on electromyography, which cannot be observed in CASPR2 associated disease (Jia et al., 2023). Thus, satellite potentials in anal sphincter electromyography indicate chronic, progressive neurodegeneration in Onuf’s nucleus.

CASPR2 can be expressed in brainstem and cerebellum. A diagnosis of CASPR2 encephalitis with cerebellar ataxia has been documented in several reports among patients positive for this antibody (Wang et al., 2021). The interpretation of a positive CASPR2-IgG result must be guided by both titer and clinical context. It is well established that high-titer antibodies (≥1:100) are highly specific for autoimmune CASPR2-associated syndromes, including encephalitis, neuromyotonia, and Morvan syndrome (Bien et al., 2017; Wang et al., 2021). However, CASPR2 antibody can also be detected in other disease involving the cerebellum (Boronat et al., 2012; Chen et al., 2024), while low-titer antibodies might represent non-specific immune responses secondary to other diseases (Boronat et al., 2012). According to prior literature, most patients with CASPR2-related diseases exhibit improvements in both clinical symptoms and cranial imaging after immunotherapy (Qin et al., 2021). Although the serum antibody titer decreased upon re-examination in this patient, further progression of brainstem and cerebellar atrophy was evident on cranial MRI. This ‘serological-clinical dissociation’ serves as a critical diagnostic marker, indicating that although the antibody was present and responsive to immunomodulatory treatment, it was likely not the primary pathogenic mechanism. In neurodegenerative disorders, high-titer autoantibodies may emerge as a secondary immune response triggered by antigen exposure due to neuronal damage, rather than representing the primary disease etiology (Chen et al., 2024). Therefore, antibody titer measurements should always be evaluated in conjunction with clinical findings. In this case, the post-treatment reduction in titer should not diminish confidence in the diagnosis of MSA-C; rather, when considered alongside the lack of clinical response, it further supports the conclusion that the underlying pathology is neurodegenerative in nature.

The presence of a high-titer CASPR2-IgG in a patient with clinically definite MSA-C raises the possibility of an underlying genetic susceptibility. It is well established that CASPR2 autoimmunity is associated with specific HLA alleles, particularly HLA-DRB1*11:01 (Binks et al., 2018; Muñiz-Castrillo et al., 2020), suggesting that an individual’s immunogenetic background may predispose them to produce this autoantibody. Although HLA genotyping was not conducted in this case—since it is not part of routine clinical practice—its potential contribution cannot be ruled out. One could speculate that such a genetic predisposition may have facilitated the production of what appears to be a clinically incidental autoantibody.

In early-stage MSA, autonomic dysfunction may remain clinically silent, making dynamic follow-up essential. EAS-EMG is high sensitivity and can detect pathological changes even before symptoms appear. This case presents a rare but informative exception, suggesting that the CASPR2 antibody was likely an incidental finding rather than the main cause of disease. It highlights a key diagnostic pitfall: even highly specific antibody results can be misleading without full clinical context. Clinical presentation, disease progression, and neuroimaging should therefore take precedence over isolated serological findings, regardless of antibody titer.

## Data Availability

The original contributions presented in the study are included in the article/supplementary material, further inquiries can be directed to the corresponding author.

## References

[ref1] Becker E. B. Zuliani L. Pettingill R. Lang B. Waters P. Dulneva A.. (2012). Contactin-associated protein-2 antibodies in non-paraneoplastic cerebellar ataxia. J. Neurol. Neurosurg. Psychiatry 83, 437–440. doi: 10.1136/jnnp-2011-301506, PMID: 22338029 PMC3297806

[ref2] Bien C. G. Mirzadjanova Z. Baumgartner C. Onugoren M. D. Grunwald T. Holtkamp M.. (2017). Anti-contactin-associated protein-2 encephalitis: relevance of antibody titres, presentation and outcome. Eur. J. Neurol. 24, 175–186. doi: 10.1111/ene.13180, PMID: 27786401

[ref3] Binks S. Varley J. Lee W. Makuch M. Elliott K. Gelfand J. M.. (2018). Distinct HLA associations of LGI1 and CASPR2-antibody diseases. Brain 141, 2263–2271. doi: 10.1093/brain/awy109, PMID: 29788256 PMC6118231

[ref4] Boronat A. Sepúlveda M. Llufriu S. Sabater L. Blanco Y. Gabilondo I.. (2012). Analysis of antibodies to surface epitopes of contactin-2 in multiple sclerosis. J. Neuroimmunol. 244, 103–106. doi: 10.1016/j.jneuroim.2011.12.023, PMID: 22245283 PMC4800093

[ref5] Boyko M. Au K. L. K. Casault C. de Robles P. Pfeffer G. (2020). Systematic review of the clinical spectrum of CASPR2 antibody syndrome. J. Neurol. 267, 1137–1146. doi: 10.1007/s00415-019-09686-2, PMID: 31912210

[ref6] Chen X. Feng L. Li J. Jiang H. (2024). Multiple system atrophy mimics CASPR2 antibody associated disease: a case report. Neurodegener. Dis. Manag. 14, 69–74. doi: 10.1080/17582024.2024.2388506, PMID: 39319563 PMC11457613

[ref7] Enomoto M. Martinez-Valbuena I. Forrest S. L. Xu X. Munhoz R. P. Li J.. (2025). Lewy-MSA hybrid fold drives distinct neuronal α-synuclein pathology. Commun. Biol. 8:929. doi: 10.1038/s42003-025-08355-7, PMID: 40523906 PMC12170825

[ref8] Fanciulli A. Wenning G. K. (2015). Multiple-system atrophy. N. Engl. J. Med. 372, 249–263. doi: 10.1056/NEJMra1311488 25587949

[ref9] Jia S. Sun C. Zhong X. Wang K. Wang Z. Qi X.. (2023). The high value of external anal-and urethral-sphincter electromyography in differential diagnosis with MSA-P, PD, and PSP. Ann. Indian Acad. Neurol. 26, 241–246. doi: 10.4103/aian.aian_496_22, PMID: 37538423 PMC10394455

[ref10] Joubert B. (2024). The neurobiology and immunology of CASPR2-associated neurological disorders. Rev. Neurol. 180, 950–956. doi: 10.1016/j.neurol.2024.09.005, PMID: 39341757

[ref11] Lim C. Y. Seo Y. Sohn B. Seong M. Kim S. T. Hong S.. (2025). The inferior cerebellar peduncle sign: a novel imaging marker for differentiating multiple system atrophy cerebellar type from spinocerebellar Ataxia. AJNR Am. J. Neuroradiol. 46, 1223–1230. doi: 10.3174/ajnr.A8623, PMID: 39674591 PMC12152783

[ref12] Masrori P. Vaesen Bentein H. Raskin J. Montagna M. De Picker L. De Volder I.. (2021). Caspr 2 autoantibody-associated Morvan syndrome predating thymoma relapse by 30 months. Lung Cancer 153, 117–119. doi: 10.1016/j.lungcan.2021.01.012 33485137

[ref13] Miao Y. Wang K. Han J. Wang Z. Bian Y. Guo Q.. (2020). Differential value of external anal-and urethral-sphincter electromyography in multiple system atrophy cerebellar type and spinocerebellar ataxias. J. Clin. Neurosci. 80, 16–22. doi: 10.1016/j.jocn.2020.07.067, PMID: 33099340

[ref14] Miki Y. Foti S. C. Asi Y. T. Tsushima E. Quinn N. Ling H.. (2019). Improving diagnostic accuracy of multiple system atrophy: a clinicopathological study. Brain 142, 2813–2827. doi: 10.1093/brain/awz189, PMID: 31289815

[ref15] Muñiz-Castrillo S. Joubert B. Elsensohn M. H. Pinto A. L. Saint-Martin M. Vogrig A.. (2020). Anti-CASPR2 clinical phenotypes correlate with HLA and immunological features. J. Neurol. Neurosurg. Psychiatry 91, 1076–1084. doi: 10.1136/jnnp-2020-323226, PMID: 32651251

[ref16] Panicker J. N. Simeoni S. Miki Y. Batla A. Iodice V. Holton J. L.. (2020). Early presentation of urinary retention in multiple system atrophy: can the disease begin in the sacral spinal cord? J. Neurol. 267, 659–664. doi: 10.1007/s00415-019-09597-2, PMID: 31720822 PMC7035234

[ref17] Qin X. Yang H. Zhu F. Wang Q. Shan W. (2021). Clinical character of CASPR2 autoimmune encephalitis: a multiple center retrospective study. Front. Immunol. 12:652864. doi: 10.3389/fimmu.2021.652864 34054814 PMC8159154

[ref18] Rubio-Agusti I. Perez-Miralles F. Sevilla T. Muelas N. Chumillas M. J. Mayordomo F.. (2011). Peripheral nerve hyperexcitability: a clinical and immunologic study of 38 patients. Neurology 76, 172–178. doi: 10.1212/WNL.0b013e3182061b1e, PMID: 21220721

[ref19] Sakakibara R. Hattori T. Uchiyama T. Kita K. Asahina M. Suzuki A.. (2000). Urinary dysfunction and orthostatic hypotension in multiple system atrophy: which is the more common and earlier manifestation? J. Neurol. Neurosurg. Psychiatry 68, 65–69. doi: 10.1136/jnnp.68.1.65, PMID: 10601404 PMC1760619

[ref21] Wang J. Qiu Z. Li D. Dong H. Hao J. Liu Z. (2021). Anti-contactin-associated protein-like 2 antibody-associated cerebellar ataxia: a case report and literature review. J. Neuroimmunol. 353:577515. doi: 10.1016/j.jneuroim.2021.577515, PMID: 33640718

[ref22] Wenning G. K. Stankovic I. Vignatelli L. Fanciulli A. Calandra-Buonaura G. Seppi K.. (2022). The Movement Disorder Society criteria for the diagnosis of multiple system atrophy. Mov. Disord. 37, 1131–1148. doi: 10.1002/mds.29005, PMID: 35445419 PMC9321158

[ref23] Yamamoto T. Sakakibara R. Uchiyama T. Yamaguchi C. Ohno S. Nomura F.. (2014). Time-dependent changes and gender differences in urinary dysfunction in patients with multiple system atrophy. Neurourol. Urodyn. 33, 516–523. doi: 10.1002/nau.22441, PMID: 23754466

